# Reproducibility of tongue pressure measurements using an experimental device

**DOI:** 10.1590/2317-1782/e20250252en

**Published:** 2026-04-27

**Authors:** Merly Fernanda Illera Castellanos, Charles Rech, Mathias Verdum de Almeida, Angela Ruviaro Busanello-Stella, Carine Cristina Callegaro

**Affiliations:** 1 Programa de Pós-graduação em Distúrbios da Comunicação Humana, Universidade Federal de Santa Maria – UFSM - Santa Maria (RS), Brasil.; 2 Laboratório de Fisiologia e Reabilitação, Departamento de Fisioterapia e Reabilitação, Universidade Federal de Santa Maria – UFSM - Santa Maria (RS), Brasil.; 3 Laboratório de Inovação Multidisciplinar – Soluções Experimentais e Computacionais, Universidade Federal de Santa Maria – UFSM - Cachoeira do Sul (RS), Brasil.; 4 Laboratório de Motricidade Orofacial, Departamento de Fonoaudiologia, Universidade Federal de Santa Maria – UFSM - Santa Maria (RS), Brasil.

**Keywords:** Tongue, Pressure, Speech-Language Pathology, Reliability, Reproducibility of Results

## Abstract

**Purpose:**

To evaluate the reproducibility of tongue pressure measurements using a low-cost experimental device in adult individuals.

**Methods:**

Ten adult volunteers of both sexes participated in the study. Tongue pressure was assessed during four distinct movements (elevation, right lateralization, left lateralization, and protrusion), with three repetitions per movement, using an experimental device. Assessments were conducted over a three-week period: the first week was dedicated to familiarization, and the subsequent two weeks to tongue pressure measurements. Reliability was analyzed using the intraclass correlation coefficient, the standard error of measurement, and the minimal detectable difference. Agreement between assessments was verified using Bland-Altman analysis.

**Results:**

The reliability of tongue pressure measurements was excellent for elevation (0.900), right lateralization (0.960), and protrusion (0.971) movements, and good for left lateralization (0.887), as demonstrated by the intraclass correlation coefficient values. The lowest standard error of measurement and minimal detectable difference were observed for right lateralization, indicating smaller measurement error. Bland–Altman analysis indicated that measurements obtained on different days showed acceptable agreement.

**Conclusion:**

Tongue pressure measurements obtained using the experimental device demonstrated reproducibility (reliability and agreement), supporting its use in clinical practice, particularly in the field of speech-language pathology.

## INTRODUCTION

The tongue is composed of extrinsic and intrinsic muscles, forming a soft tissue located in the oral cavity^(1)^. This muscle group acts as a dynamic biomechanical system, allowing changes in position and shape, which is essential for functions such as speech, breathing, and swallowing^(2)^. These are affected by atrophy and weakness of the tongue muscles^(3)^, predisposing to dysarthria^(4)^, obstructive sleep apnea (OSA)^(5)^, and dysphagia^(6)^.

Clinical practice widely uses subjective protocols to assess tongue muscle tone, a fundamental aspect for its mobility, coordination, and resistance^(7)^. However, the objective measurement of tongue pressure against a sensor in protrusion, elevation, and lateral displacement movements^(8)^ provides more accurate data to be used in speech-language-hearing (SLH) therapy and enables the monitoring of clinical evolution and the direction of therapeutic approaches. Tongue pressure has been measured using specific equipment, such as the Iowa Oral Performance Instrument (IOPI) and the JMS TPM-01 system, widely recognized for their accuracy and ease of use^(9)^. The Tongueometer has also been used, standing out for its Bluetooth technology, which allows real-time assessment through an electronic device^(10)^. The Biofeedback Pró-Fono: Lip and Tongue Pressure device is available on the Brazilian market, being used in clinical and research contexts for measuring orofacial strength^(11)^. These devices eliminate the subjectivity of manual methods^(7)^, making the measurement more reliable. However, their high cost^(12)^ hinders adoption in SLH clinics and restricts the population's access to SLH assessments and therapies based on objective data.

In addition to commercial equipment, national initiatives have sought to develop more accessible alternatives. One example is the experimental device developed by researchers at the Federal University of Minas Gerais, Brazil, which demonstrated good reproducibility in assessing tongue axial force, although further studies are still needed for standardization and large-scale validation^(13)^. However, investigations reporting the reliability, agreement, and measurement error of available instruments are scarce. The absence of this methodological standardization limits comparability between studies and restricts the clinical applicability of the findings. Hence, it is a priority to develop and validate devices that, in addition to being accessible, present consistent metrological performance and reproducible measurements. Accordingly, aiming to expand access to objective assessment of tongue pressure in resource-limited settings, our group developed a portable, low-cost device with a high-resolution sensor (uncertainty ± 1%). Preliminary mechanical tests indicated adequate stability and resolution^(14)^; however, its clinical applicability in adults still needs to be demonstrated. Thus, this study aimed to evaluate the reproducibility (reliability and agreement) of tongue pressure measurements in adults using this low-cost experimental device.

## METHODS

This is a cross-sectional study of the reliability and agreement of tongue pressure measurements, approved by the Research Ethics Committee of a public university under approval number 6.770.205. All participants signed an informed consent form.

The study was conducted with 10 volunteers (six men and four women), with a mean age of 31.3 ± 6.3 years (minimum of 26 and maximum of 45 years) and a mean body mass index (BMI) of 24.5 ± 2.4 kg/m^2^.

All volunteers presented scores on the Orofacial Myofunctional Evaluation Protocol with Scores-extended (OMES-E) compatible with Brazilian reference values ​​for normality (score greater than 152), suggesting the absence of orofacial myofunctional disorder (OMD)^(15)^.

The participants were professionals with completed higher education, or students in the final stages of their undergraduate, master's, or doctoral studies at the university where the study was conducted. None of the participants reported a history of dysphagia or sleep-related complaints. All had 28 to 32 permanent teeth, with occlusal contact in the premolar and molar regions, without the use of prostheses or orthodontic appliances, and without a report of periodontal disease.

The initial assessment included an interview to collect the clinical history and orofacial myofunctional examination based on a clinical protocol^(15)^, including tests of facial pattern and oral cavity analysis to exclude individuals with abnormal lingual frenulum that limited tongue movements.

Participants with a history and/or signs suggestive of central or peripheral neurological disorders, head and neck surgeries, tumors, or traumas, and who had undergone previous SLH therapy for orofacial strengthening were excluded.

**Figure 1:**
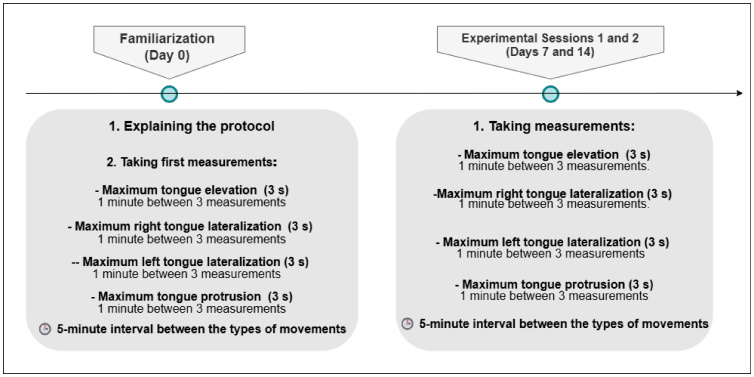
Descriptive diagram of the tongue pressure assessment procedure. Experimental sessions 1 and 2 (days 7 and 14) followed the same protocol as the familiarization session, with a 5-minute interval between the different types of movements

First, participants became familiarized with the protocol. Then, they made two visits to the laboratory for the experimental sessions, with a 7-day interval between them (days 7 and 14). Both sessions rigorously followed the same evaluation protocol as the familiarization session, including the same rest times between repetitions (1 minute) and between different types of movement (5 minutes), as illustrated in Figure 1. Each maximum pressure effort was sustained for 3 seconds in each repetition. All participants were evaluated by the same examiner, who also conducted both test sessions, ensuring a standardized procedure. OMES-E was applied at a different time, before the tongue pressure measurements, to characterize the orofacial myofunctional profile of the sample before performing the experimental tests.

### Tongue pressure assessment

**Figure 2:**
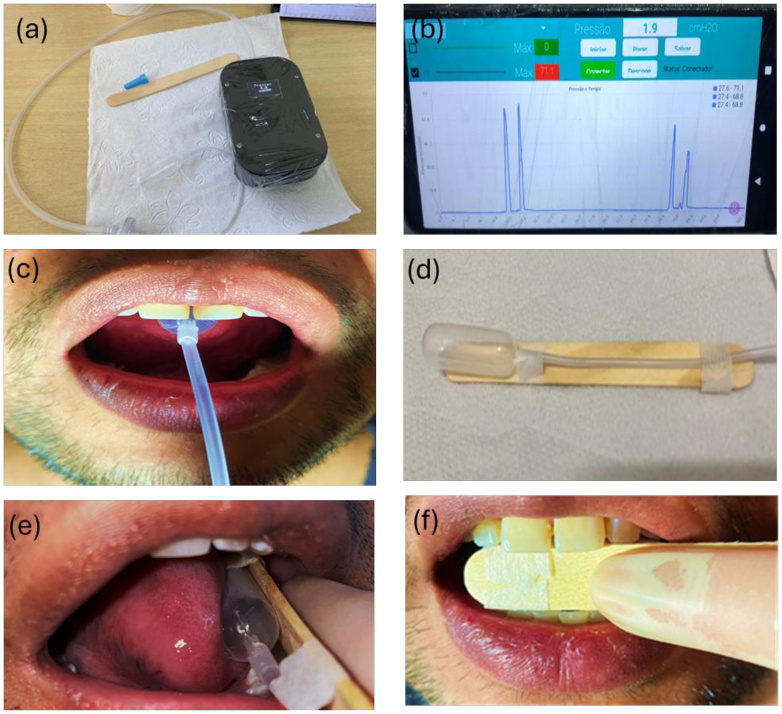
Experimental device for measuring tongue pressure and its application. (a) Experimental device for measuring tongue pressure; (b) Real-time tongue pressure recorded by the system; (c) Measurement of tongue elevation pressure, with the bulb positioned longitudinally along the hard palate, near the incisive papilla; (d) Bulb attached to the spatula; (e) Bulb positioning for measuring tongue lateralization; (f) Bulb positioning for measuring tongue protrusion

Tongue pressure measurements were taken using an experimental device^(14)^ developed for the objective evaluation of this function. Figure 2a shows the complete set of the device used. The system consists of a piezoresistive pressure sensor (model XGZP6847100KPG), with a measurement range of 0 to 100 kPa, a resolution of 0.025 kPa, and an uncertainty of ± 1%. This sensor is connected to an ADS1115 (16-bit) analog-to-digital converter and an ESP-WROOM-32 microcontroller, which processes and transmits data via Bluetooth (Figure 2b). The air bulb, coupled to the sensor by a 3 mm flexible tube, was positioned in the oral cavity according to the evaluated movement (elevation, lateralization, or protrusion). The system's stability, sampling rate, and circuit resolution ensured sufficient accuracy for recording maximum pressure during isometric exercises. The procedure followed the protocol described by Clark et al.^(8)^ and is illustrated in the remaining parts of Figure 2.

Tongue elevation: only the tip of the tongue was used to measure maximum pressure, with the bulb positioned longitudinally along the hard palate, just after the superior alveolar crest, close to the incisive papilla. Participants were instructed to raise the tip of their tongue and press it against the bulb with maximum effort and maintain maximum pressure for 3 seconds on each attempt (Figure 2c).

Tongue lateralization: The bulb was attached to a wooden spatula fixed with adhesive tape (Figure 2d), positioned between the posterior teeth to provide stable support, as described by Clark et al.^(8)^. Volunteers were instructed not to clench their teeth against the spatula, ensuring that the bulb did not come into direct contact with the teeth. With the mouth slightly open, the bulb was positioned in the region of the premolar and molar teeth. Participants were instructed to push the side of their tongue against the bulb with maximum pressure, performing separate measurements, starting on the right side and then on the left side (Figure 2e). Each attempt lasted 3 seconds, with three repetitions for each side.

Tongue protrusion: This used the same spatula stabilization procedure as in lateralization, but with the bulb positioned in the region of the lingual surface of the incisor teeth (Figure 2f). Participants were instructed to project their tongue against the bulb with maximum possible effort and to maintain pressure for 3 seconds in each attempt.

The examiner gave verbal commands such as "push," "push!", "squeeze, squeeze, squeeze!". All measurements were supervised to ensure data accuracy, and the device was recalibrated between tests.

The tests were performed with participants seated, with their backs supported, feet firmly on the ground, and their heads maintained in a neutral posture. Each tongue pressure measurement was taken with a 1-minute rest interval^(16)^ between repetitions to avoid muscle fatigue. The examiner noted the maximum pressure recorded in each repetition; the highest of the three measurements was considered for analysis. They also had a 5-minute interval between the different types of movement to minimize accumulated fatigue and possible interference between efforts. The pressure exerted by the tongue was measured in centimeters of water (cmH_2_O) and then converted to kilopascals (kPa), using the conversion factor (1 cmH_2_O = 0.098 kPa).

To ensure biosecurity, the pressure measuring device and the cell phone were sanitized before each use, and both were wrapped with disposable film paper, which was replaced at each session.

### Statistical analysis

The sample size was calculated using the F-test, considering the detection of a difference between a null ICC of 0.50 and an expected ICC of 0.75, with a 5% significance level and 80% statistical power. The estimated minimum was nine subjects with two measurements per participant. The calculation was performed in the R software, using the pwr package. Data were analyzed using descriptive statistics, presenting OMES-E variables as means and standard deviations and the variables with non-parametric distribution as median and interquartile range (IQR). The Wilcoxon test for paired samples compared tongue pressure measurements between weeks 1 and 2, appropriate for data with a non-parametric distribution. P < 0.05 was considered significant.

Reliability (repeatability and reproducibility) was assessed with the intraclass correlation coefficient (ICC 3.1), the standard error of measurement (SEM), and the minimal detectable change (MDC). The ICC 3.1 was used because it is suitable for mixed models of two-way analysis of variance, where the effects of individuals are considered random, and those of the measurements are fixed. This model assesses the absolute agreement of single measurements. The ICC was classified as described by Koo and Li^(17)^, as low agreement (< 0.50), moderate agreement (0.50–0.75), good agreement (0.75–0.90), and excellent agreement (> 0.90). A 95% confidence interval (CI) was calculated for the ICC.

The SEM was calculated to quantify the measurement imprecision, using the Formula 1:


$$\;SEM\;=\;combined\;SD\times \;\sqrt{1-ICC}$$ (1)

in which the combined SD was obtained by averaging the SDs of the two weeks, calculated as (Formula 2):


$$combined\;SD=\frac{\sqrt{SD^{2}week\;1+\;SD^{2}week\;2}}{2}$$ (2)

The MDC was calculated to determine the smallest distinguishable change in measurement error^(18)^, using the Formula 3:


$$MDC=1.96\;\times \;\sqrt{2}\times SEM$$ (3)

Bland-Altman plots were used to assess the agreement between the measurements of the two weeks, representing the individual differences (y-axis) in relation to the means observed between the assessments (x-axis). The 95% limits of agreement were calculated using the equation: mean difference ± (1.96 × SD of differences)^(19)^. A significance level of p < 0.05 was considered. All statistical analyses were conducted using SPSS software (version 27, IBM Corp., Armonk, NY, USA).

## RESULTS

Participants had high orofacial functioning, with a total mean score of 222.4 ± 5.48 points, corresponding to 95.86% of the maximum possible score (232 points). All individuals obtained total scores above the cutoff established by OMES-E (152 points), with estimated values ​​ranging from 217 to 228 points. In all evaluated domains, 100% of participants had scores within the normal range (≥ 50%), with particular emphasis on Mobility (98.86% of the maximum score) and Function (96.3%). Greater variability was observed in Mastication (SD = 1.78) and Mandible – Appearance (SD = 1.83), reflecting expected individual differences. These findings indicate preserved orofacial functioning in the study sample, as presented in Table 1.

**Table 1:** Orofacial Myofunctional Assessment

**Item and Category**	**Maximum score**	**Scores (N = 10)** **Mean ± SD**	**Min – Max**	**% of the maximum score**
Appearance/Posture	64	57.7 ± 3.95	53.75 – 61.65	90.16%
Face	12	9.8 ± 1.23	8.57 – 11.03	81.67%
Cheeks	8	7.6 ± 0.52	7.08 – 8.12	95.00%
Mandible	12	10±1.83	8.17 – 11.83	83.33%
Lips	16	10.9 ±1.1	9.80 – 12.00	68.12%
Palate	8	7.8 ±0.42	7.38 – 8.22	97.50%
Tongue	8	7.9 ±0.32	7.58 – 8.22	98.75%
Mobility	114	112.7 ±1.7	111.00-114.40	98.86%
Lips	24	23.7 ±0.67	23.03 – 24.37	98.75%
Tongue	36	36 ±0	36.00	100.00%
Mandible	30	29.4 ± 1.26	28.14 – 30.66	98.00%
Cheeks	24	23.6 ± 1.26	22.34 – 24.86	98.33%
Functions	54	52 ± 2. 26	49.74 – 54.26	96.30%
Breathing	4	3.9 ± 0.32	3.58 – 4.22	97.50%
Swallowing	28	27.6 ± 0.7	26.90 – 28.30	98.57%
Chewing	22	20.5 ±1.78	18.72 – 22.28	93.18%
Total OMES-E	232	222.4 ± 5.87	216.92 – 227.88	95.86%

**Caption:** OMES-E = Orofacial Myofunctional Evaluation Protocol with Scores-extended; N = Number of participants; SD = standard deviation; Min = minimum; Max = maximum; % = percentage calculated in relation to the maximum possible score per category/domain

Table 2 simultaneously presents the medians (IQR) of weeks 1 and 2 and the intrarater reliability indices (ICC, SEM, and MDC) for the four movements. Only right lateralization increased significantly between weeks (p = 0.047), while elevation, left lateralization, and protrusion did not differ (p ≥ 0.169). Reliability ranged from good to excellent (ICC = 0.887–0.971). SEM and MDC were lower in right lateralization (SEM = 0.86; MDC = 2.38) and higher in left lateralization (SEM = 1.55; MDC = 4.30), indicating greater accuracy for right lateralization.

**Table 2:** Tongue pressure measurements (weeks 1 and 2) and intrarater reliability (N = 10)

**Movements**	**Week 1 Median (IQR) kPa**	**Week 2 Median (IQR) kPa**	**p-value**	**ICC (95% CI)**	**SEM**	**MDC**	**p-value**
**Elevation**	21.76	23.21	0.169	0.900	1.45	4.05	< 0.001
	(0.01–26.19)	(21.99–25.39)		(0.623–0.975)			
**R Lateralization**	14.42	14.86	0.047*	0.960	0.86	2.38	< 0.001
	(12.06–19.43)	(13.26–20.58)		(0.803–0.990)			
**L Lateralization**	17.39	16.99	0.799	0.887	1.55	4.30	0.002
	(12.90–22.80)	(13.98–20.94)		(0.527–0.972)			
**Protrusion**	26.32	27.85	0.285	0.971	1.02	2.83	< 0.001
	(24.11–31.39)	(23.75–32.78)		(0.889–0.993)			

Wilcoxon test

* Significant values ​​at P < 0.05

**Caption:** IQR = Interquartile Range; kPa = kilopascal; ICC = Intraclass Correlation Coefficient; 95% CI = 95% Confidence Interval; SEM = Standard Error of Measurement; MDC = Minimal Detectable Change

**Figure 3:**
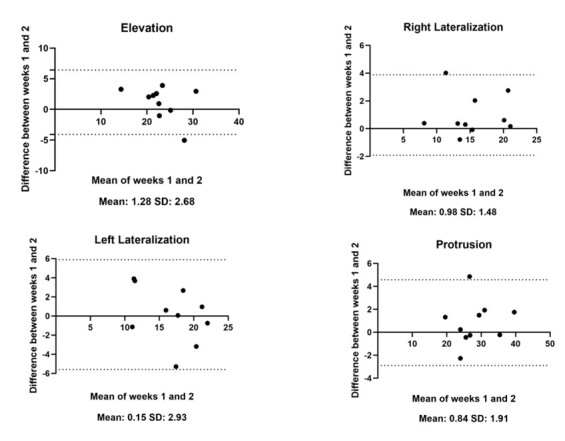
Graphical representations of the Bland–Altman diagram for tongue pressure measurements during elevation, right lateralization, left lateralization, and protrusion

Figure 3 shows good agreement between weeks 1 and 2 for all movements, with mean differences close to zero, through Bland-Altman analysis. Greater variability was observed in elevation (mean difference: 1.18 kPa, SD: 2.68) and protrusion (mean difference: 0.84 kPa, SD: 1.91), while right lateralization had less variability (mean difference: 0.98 kPa, SD: 1.48), and left lateralization had greater dispersion (mean difference: 0.15 kPa, SD: 2.93).

## DISCUSSION

This study evaluated the reproducibility of tongue pressure measurements in adults using an experimental low-cost device. Intrarater reliability was good to excellent, suggesting that the measurements can be used in clinical practice.

The device used in this study has good stability and allows real-time recording, facilitating evaluation and feedback for the participant. It was developed with low-cost components that are readily available on the national market. Details of the development and estimated cost (approximately US$ 49) are described in Almeida et al.^(14)^, which suggests its viability for clinical and home use, including in resource-limited settings.

This device is more versatile than traditional devices, such as the IOPI, widely used for measuring tongue pressure. The latter is traditionally used to assess elevation pressure^(20)^, but studies indicate that it can be adapted for lateralization measurements, expanding its applicability^(16,21)^. In this study, we followed the procedures with the IOPI described by Clark et al.^(8)^, performing elevation, lateralization, and protrusion measurements (with the bulb attached to the spatula for directional measurements), which allows a more comprehensive analysis of lingual functioning^(8)^. In the present study, the ICCs for tongue pressure were similar to those previously reported^(13,22)^, ranging from 0.72 to 0.97. These findings reinforce the reliability and reproducibility of objective devices in the assessment of lingual pressure. The study by Diaz-Saez et al.^(22)^ with the Spanish population evaluated a device for measuring tongue pressure and reported good to excellent intrarater and interrater reliability, with ICC values ​​greater than 0.80, reinforcing the viability of using these instruments in clinical practice. Araújo et al.^(13)^ analyzed the reproducibility of the Forling, a portable instrument developed by Brazilian researchers to measure tongue axial strength, and observed acceptable ICCs, as well as variation within the desirable margin, indicating its reliability for clinical and research use. Although most measurements in this study remained unchanged between tests, right lateralization increased significantly in the second week. As participants underwent a prior familiarization process, this difference may be associated with motor adjustments and neuromuscular refinement throughout the experimental sessions^(23)^. Neuroplasticity allows adjustments in motor control, favoring motor learning^(24)^. Studies indicate that repetitive tongue movements can induce neuroplasticity and improve intermuscular coordination^(25)^. The study by Kamarunas et al.^(23)^ highlights that the optimization of motor recruitment and coordination can increase muscle strength. This effect could explain the improvement in right lateralization, aligning with the findings of Araújo et al.^(13)^, who observed a progressive increase in strength throughout the measurements, also attributing it to adaptation to the assessment procedure, especially between the first and second experimental sessions.

Intrarater reliability was good to excellent, demonstrating consistency between weeks 1 and 2. However, left lateralization had greater variability, possibly due to an individual with outlier values, as evidenced by the Bland-Altman analysis. This individual was identified in the OMES-E, and some alterations in items such as mastication and mandibular relationship may have contributed to the variation in tongue force between the first and second experimental sessions (Figure 3). This suggests that structural or functional factors, such as dental occlusion and the temporomandibular joint (TMJ), may influence tongue movements. The study by Yu and Gao^(26)^ demonstrated that the distribution of tongue pressure is related to the shape of the dental arch and occlusion. The authors observed that tongue pressure against the palate is influenced by the width and length of the dental arch, impacting bite stability and orofacial functioning. Moreover, Diaz-Saez et al.^(27)^ identified significant differences in tongue pressure between asymptomatic women and those with temporomandibular dysfunction (TMD), highlighting the relevance of this clinical assessment. In this study, a mean reduction of approximately 30% in tongue pressure was observed in all directions in the TMD group, reinforcing the need to consider tongue pressure in clinical practice. The results of this study showed that tongue pressure measurements were lower than those reported in research using commercial devices^(9,10)^. This difference may be associated with the particularities of the devices, which vary in sensitivity, calibration, and contact area, and the characteristics of the population evaluated. Studies indicate that the reported values ​​may be influenced by the measurement method, sensor positioning, and measurement protocol^(28)^. Factors such as sex, age, and familiarity with the test may also impact the results, with men tending to have higher tongue pressure, while lateralization and protrusion movements are less explored in the literature^(29)^.

The stability of the sensor can also affect the measurement of tongue pressure, especially in lateralization and protrusion movements. Solomon and Clark^(30)^ demonstrated that adding a non-slip surface to the sensor resulted in significantly higher maximum pressure values ​​in these directions, suggesting that tongue slippage can compromise the accuracy of the results. This highlights the importance of considering the type of support used in measuring tongue pressure, especially in comparative studies between different devices and methodologies.

This study has some limitations. It evaluated only the maximum pressures of elevation, lateralization, and protrusion, not including the maximum posterior pressure, which restricts a complete view of tongue functioning in different directions of movement. Moreover, the sample consisted exclusively of healthy adults, which does not allow direct extrapolation of the results to populations with orofacial changes or specific clinical conditions. Future studies should consider different directions of movement and include clinical groups to broaden the generalizability of the findings.

The study findings may impact the clinical practice of orofacial motor skills by demonstrating that the experimental device is an accessible, low-cost tool for assessing tongue pressure. Future studies should investigate its applicability in different clinical contexts, expanding the possibilities for its use.

## CONCLUSION

This study demonstrated that measuring tongue pressure with the proposed device in adults has good to excellent reproducibility. Protrusion was the movement with the highest accuracy, while left lateralization had greater variability, possibly due to structural and functional factors. The high intrarater reliability indicates that the device can be useful for objective assessments of tongue pressure. Furthermore, its low cost favors its incorporation into SLH practice, expanding the possibilities for clinical assessment and monitoring.
