# Supplementary material for: Reproducibility of tongue pressure measurements using an experimental device
Source: Codas. 2026 Apr 27;38(3):e20250252. doi: 10.1590/2317-1782/e20250252en (PMC13193197; doi:10.1590/2317-1782/e20250252en)
Supplement: Translation

# Translation: Reprodutibilidade das medidas de pressão da língua utilizando um dispositivo experimental

**DOI:** 10.1590/2317-1782/e20250252pt

**Authors:** Merly Fernanda Illera Castellanos, Charles Rech, Mathias Verdum de Almeida, Angela Ruviaro Busanello-Stella, Carine Cristina Callegaro

**Affiliations:** 1 Programa de Pós-graduação em Distúrbios da Comunicação Humana, Universidade Federal de Santa Maria – UFSM - Santa Maria (RS), Brasil.; 2 Laboratório de Fisiologia e Reabilitação, Departamento de Fisioterapia e Reabilitação, Universidade Federal de Santa Maria – UFSM - Santa Maria (RS), Brasil.; 3 Laboratório de Inovação Multidisciplinar – Soluções Experimentais e Computacionais, Universidade Federal de Santa Maria – UFSM - Cachoeira do Sul (RS), Brasil.; 4 Laboratório de Motricidade Orofacial, Departamento de Fonoaudiologia, Universidade Federal de Santa Maria – UFSM - Santa Maria (RS), Brasil.

**Keywords:** Língua, Pressão, Fonoaudiologia, Confiabilidade, Reprodutibilidade dos Testes

## Abstract

**Objetivo:**

Avaliar a reprodutibilidade das medidas de pressão da língua utilizando um dispositivo experimental e de baixo custo em indivíduos adultos.

**Método:**

Dez voluntários, adultos de ambos os sexos, participaram do estudo. A pressão da língua foi avaliada em quatro movimentos distintos (elevação, lateralização direita, lateralização esquerda e protrusão), com três repetições de cada movimento, utilizando um dispositivo experimental. Essas medições foram realizadas ao longo de três semanas: a primeira foi destinada à familiarização e as duas semanas seguintes às mensurações da pressão da língua. A confiabilidade foi analisada por meio do coeficiente de correlação intraclasse, erro padrão da medição e diferença mínima detectável. A concordância entre as avaliações foi verificada por meio da análise de Bland-Altman.

**Resultados:**

A confiabilidade das medidas de pressão da língua foi excelente para os movimentos de elevação (0,900), lateralização direita (0,960) e protrusão (0,971) e boa para a lateralização esquerda (0,887), conforme demonstrado pelos valores do coeficiente de correlação intraclasse. O menor erro-padrão da medida e a menor diferença mínima detectável foram observados na lateralização direita, indicando menor erro de medição. A análise de Bland-Altman sugere que as medidas em dias diferentes apresentam concordância aceitável.

**Conclusão:**

As medidas de pressão da língua, utilizando o dispositivo experimental apresentam reprodutibilidade (confiabilidade e concordância), possibilitando sua aplicação na prática clínica, especialmente no contexto fonoaudiológico.

## INTRODUÇÃO

A língua é composta por músculos extrínsecos e intrínsecos, formando um tecido mole situado na cavidade oral^(1)^. Esse conjunto muscular atua como um sistema biomecânico dinâmico, permitindo mudanças na posição e no formato, o que é essencial para funções como fala, respiração e deglutição^(2)^, as quais são afetadas pela atrofia e fraqueza dos músculos da língua^(3)^, predispondo a ocorrência de disartria^(4)^, apneia obstrutiva do sono (AOS)^(5)^ e disfagia^(6)^.

Na prática clínica, protocolos subjetivos são amplamente utilizados para avaliar o tônus muscular da língua, um aspecto fundamental para sua mobilidade, coordenação e resistência^(7)^. Entretanto, a mensuração objetiva da pressão exercida pela língua contra um sensor, nos movimentos de protrusão, elevação e deslocamento lateral^(8)^, fornece dados mais precisos para serem utilizados na atuação fonoaudiológica, além de possibilitar o acompanhamento da evolução clínica e o direcionamento das condutas terapêuticas. A pressão da língua tem sido mensurada por meio de equipamentos específicos, como o *Iowa Oral Performance Instrument* (IOPI) e o sistema JMS TPM-01, amplamente reconhecidos pela precisão e facilidade de uso^(9)^. Além desses, o *Tonguemeter* também tem sido utilizado, destacando-se pelo uso de tecnologia *Bluetooth*, que permite a avaliação em tempo real por meio de um dispositivo eletrônico^(10)^. No Brasil, encontra-se disponível no mercado, o equipamento *Biofeedback Pró-Fono*: *Pressão de Lábios e de Língua*, utilizado em contextos clínicos e de pesquisa para a mensuração da força orofacial^(11)^. Esses equipamentos eliminam a subjetividade de métodos manuais^(7)^, tornando a mensuração mais confiável. No entanto, seu elevado custo^(12)^ dificulta a adoção em clínicas de Fonoaudiologia e restringe o acesso da população a avaliações e terapias fonoaudiológicas baseadas em dados objetivos.

Além dos equipamentos comerciais, iniciativas nacionais têm buscado desenvolver alternativas mais acessíveis. Um exemplo é o dispositivo experimental desenvolvido por pesquisadores da Universidade Federal de Minas Gerais, que demonstrou boa reprodutibilidade na avaliação da força axial da língua, embora ainda sejam necessários estudos adicionais para padronização e validação em larga escala^(13)^. Observa-se, porém, escassez de investigações que relatem a confiabilidade, a concordância e o erro de medição dos instrumentos disponíveis. A ausência dessa padronização metodológica limita a comparabilidade entre estudos e restringe a aplicabilidade clínica dos achados. Nesse contexto, torna-se prioritário desenvolver e validar dispositivos que, além de acessíveis, apresentem desempenho metrológico consistente e medidas reprodutíveis. No mesmo sentido, visando ampliar o acesso à avaliação objetiva da pressão lingual em contextos com recursos limitados, nosso grupo desenvolveu um dispositivo portátil e de baixo custo, com sensor de alta resolução (incerteza ±1%). Ensaios mecânicos preliminares indicaram estabilidade e resolução adequadas^(14)^; entretanto, sua aplicabilidade clínica em adultos ainda precisa ser demonstrada. Assim, o objetivo deste estudo foi avaliar a reprodutibilidade (confiabilidade e concordância) das medidas de pressão da língua em indivíduos adultos utilizando esse dispositivo experimental de baixo custo.

## MÉTODO

Trata-se de um estudo transversal de confiabilidade e concordância das medidas da pressão da língua, aprovado pelo Comitê de Ética em Pesquisa de uma universidade pública, conforme parecer número 6.770.205. Todos os participantes assinaram o Termo de Consentimento Livre e Esclarecido.

O estudo foi desenvolvido com dez voluntários (seis homens e quatro mulheres), com média de idade de 31,3 ± 6,3 anos (mínima de 26 e máxima de 45 anos) e índice de massa corporal (IMC) médio de 24,5 ± 2,4 kg/m^2^.

Todos os voluntários apresentaram pontuação no Protocolo de Avaliação Miofuncional Orofacial com Escores Expandido (AMIOFE-E) compatível com os valores de referência brasileiros para normalidade (escore superior a 152), sugerindo ausência de disfunção miofuncional orofacial (DMO)^(15)^.

Os participantes eram profissionais com ensino superior completo, ou estudantes em fase de conclusão, cursando graduação, mestrado ou doutorado na universidade onde o estudo foi realizado. Nenhum dos participantes relatou histórico de disfagia ou queixas relacionadas ao sono. Todos possuíam entre 28 e 32 dentes permanentes, com contato oclusal nas regiões de pré-molares e molares, sem uso de próteses, aparelhos ortodônticos e sem relato de doença periodontal.

A avaliação inicial incluiu entrevista para coleta da história clínica e exame miofuncional orofacial baseado em protocolo clínico^(15)^, incluindo provas de análise do padrão facial e da cavidade oral, com o objetivo de excluir indivíduos com alterações do frênulo lingual que limitassem os movimentos da língua.

Foram excluídos participantes com histórico e/ou sinais sugestivos de distúrbios neurológicos centrais ou periféricos, cirurgias, tumores ou traumas na região da cabeça e pescoço, bem como aqueles que haviam realizado terapia fonoaudiológica prévia para fortalecimento orofacial.

Primeiramente, os participantes foram familiarizados em relação ao protocolo, após realizaram duas visitas ao laboratório para as sessões experimentais, com intervalo de 7 dias entre elas (dias 7 e 14). Ambas as sessões seguiram rigorosamente o mesmo protocolo de avaliação da sessão de familiarização, incluindo os mesmos tempos de repouso entre repetições (1 minuto) e entre os diferentes tipos de movimento (5 minutos), conforme ilustrado na Figura 1. Cada esforço de pressão máxima foi sustentado por 3 segundos em cada repetição. Todos os participantes foram avaliados pela mesma examinadora, que também conduziu ambas as sessões de teste, garantindo a padronização do procedimento. Importante destacar que a aplicação do protocolo AMIOFE-E foi realizada em um momento distinto e anterior às medições da pressão da língua, a fim de caracterizar o perfil miofuncional orofacial da amostra antes da execução dos testes experimentais.

**Figura 1:** Esquema descritivo do procedimento de avaliação da pressão da língua. As sessões experimentais 1 e 2 (dias 7 e 14) seguiram o mesmo protocolo da sessão de familiarização, com intervalo de cinco minutos entre os diferentes tipos de movimento

### Avaliação da pressão da língua

As medições de pressão da língua foram realizadas com o auxílio de um dispositivo experimental^(14)^, desenvolvido para avaliação objetiva dessa função. A Figura 2a apresenta o conjunto completo do dispositivo utilizado. O sistema é composto por um sensor de pressão piezoresistivo (modelo XGZP6847100KPG), com faixa de medição de 0 a 100 kPa, resolução de 0,025 kPa e incerteza de ±1%. Esse sensor é conectado a um conversor analógico-digital ADS1115 (16 bits) e a um microcontrolador ESP-WROOM-32, que realiza o processamento e a transmissão dos dados via Bluetooth (Figura 2b). O bulbo de ar, acoplado ao sensor por um tubo flexível de 3 mm, foi posicionado na cavidade oral conforme o movimento avaliado (elevação, lateralização ou protrusão). A estabilidade do sistema, a taxa de amostragem e a resolução do circuito garantiram precisão suficiente para os registros de pressão máxima durante os exercícios isométricos. O procedimento seguiu o protocolo descrito por Clark et al.^(8)^ e está ilustrado nas demais partes da Figura 2.

**Figura 2:** Dispositivo experimental para medição da pressão da língua e sua aplicação. (a) Dispositivo experimental para medir a pressão da língua; (b) Pressão da língua em tempo real registrada pelo sistema; (c) Medida de pressão da elevação da língua, com o bulbo posicionado longitudinalmente ao longo do palato duro, próximo à papila incisiva; (d) Bulbo acoplado à espátula; (e) Posicionamento do bulbo para medição da lateralização da língua; (f) Posicionamento do bulbo para medição da protrusão da língua

Elevação da língua: Para mensurar a pressão máxima, foi utilizada apenas a ponta da língua, com o bulbo posicionado longitudinalmente ao longo do palato duro, logo após a crista alveolar superior, próximo à papila incisiva. Os participantes foram instruídos a elevar a ponta da língua e pressioná-la contra o bulbo com o máximo esforço possível e a manter a pressão máxima por três segundos em cada tentativa (Figura 2c).

Lateralização da língua: O bulbo foi acoplado a uma espátula de madeira fixado com fita adesiva (Figura 2d), posicionado entre os dentes posteriores para fornecer suporte estável, conforme descrito por Clark et al.^(8)^. Os voluntários foram orientados a não apertar os dentes contra a espátula, garantindo que o bulbo não entrasse em contato direto com os dentes. Com a boca levemente aberta, o bulbo foi posicionado na região dos dentes pré-molares e molares. Os participantes foram instruídos a empurrar a lateral da língua contra o bulbo com pressão máxima, realizando medições separadas, iniciando pelo lado direito e, em seguida, pelo lado esquerdo (Figura 2e). Cada tentativa teve duração de três segundos, com três repetições para cada lado.

Protrusão da língua: O mesmo procedimento de estabilização da espátula utilizado na lateralização foi adotado, mas com o bulbo posicionado na região da superfície lingual dos dentes incisivos (Figura 2f). Os participantes foram orientados a projetar a língua contra o bulbo com o máximo esforço possível e a manter também a pressão por três segundos em cada tentativa.

A examinadora forneceu comandos verbais como "pressiona", "pressiona!", "aperta, aperta, aperta!". Todas as medições foram supervisionadas para garantir a precisão dos dados, e o dispositivo foi recalibrado entre cada teste.

Os testes foram realizados com os participantes sentados, com as costas apoiadas, pés firmes no chão e a cabeça mantida em postura neutra. Cada medição da pressão da língua foi realizada com um intervalo de descanso de um minuto^(16)^ entre cada repetição para evitar fadiga muscular. A pressão máxima registrada em cada repetição foi anotada pela examinadora, sendo considerada para análise a maior das três medições. Entre os diferentes tipos de movimento, foi adotado um intervalo de cinco minutos, a fim de minimizar o cansaço acumulado e possíveis interferências entre os esforços. A pressão exercida pela língua foi mensurada em centímetros de água (cmH_2_O) e posteriormente convertida para kilopascais (kPa), utilizando o fator de conversão (1 cmH_2_O = 0,098 kPa).

Para garantir a biossegurança, o dispositivo de medida de pressão e o celular foram higienizados antes de cada uso e ambos foram envolvidos com papel filme descartável, substituído a cada sessão.

### Análise estatística

O tamanho da amostra foi calculado utilizando o teste F, considerando a detecção de uma diferença entre um ICC nulo de 0,50 e um ICC esperado de 0,75, com nível de significância de 5% e poder estatístico de 80%. Com duas medições por participante, o número mínimo estimado foi de 9 sujeitos. O cálculo foi realizado no software R, utilizando o pacote pwr. Os dados foram analisados utilizando estatísticas descritivas, com as variáveis do protocolo AMIOFE-E apresentadas como médias e desvios padrão, e as variáveis com distribuição não paramétrica expressas como mediana e intervalo interquartil (IQR). Para a comparação das medidas de pressão da língua entre as semanas 1 e 2, foi aplicado o teste de Wilcoxon para amostras pareadas, apropriado para dados com distribuição não paramétrica. Considerou-se como significativo p < 0,05.

A confiabilidade (repetibilidade e reprodutibilidade) foi avaliada utilizando o Coeficiente de Correlação Intraclasse (ICC 3,1), o Erro Padrão da Medição (EPM) e a Diferença Mínima Detectável (DMD). O ICC 3,1 foi utilizado por ser adequado para modelos mistos de análise de variância bidirecional, onde os efeitos das pessoas são considerados aleatórios e os das medidas, fixos. Esse modelo avalia a concordância absoluta de medidas únicas. O ICC foi classificado conforme descrito por Koo e Li^(17)^, como: baixa concordância (<0,50), moderada concordância (0,50–0,75), boa concordância (0,75–0,90) e excelente concordância (>0,90). Um intervalo de confiança (IC) de 95% foi calculado para o ICC.

O EPM foi calculado para quantificar a imprecisão da medição, utilizando a Fórmula 1:


$$\;EPM\;=\;DP\;combinado\times \;\sqrt{1-ICC}$$ (1)

onde o desvio padrão combinado (DP *combinado)* foi obtido pela média dos desvios padrão das duas semanas, calculado como (Fórmula 2):


$$DP\;combinado=\frac{\sqrt{DP^{2}semana\;1+\;DP^{2}semana\;2}}{2}$$ (2)

A DMD foi calculada para determinar a menor mudança distinguível do erro de medição^(18)^, utilizando a Fórmula 3:


$$MDC\;=\;1.96\;\times \;\sqrt{2}\times EPM$$ (3)

Para avaliar a concordância entre as medições das duas semanas, foram utilizados gráficos de Bland-Altman, que representam as diferenças individuais (eixo y) em relação às médias observadas entre as avaliações (eixo x). Os limites de concordância de 95% foram calculados pela equação: diferença média ± (1,96 × desvio padrão das diferenças)^(19)^. Considerou-se nível de significância de p < 0,05. Todas as análises estatísticas foram conduzidas no software SPSS (versão 27, IBM Corp., Armonk, NY, EUA).

## RESULTADOS

Os participantes apresentaram alta funcionalidade orofacial, com pontuação média total de 222,4 ± 5,48 pontos, o que corresponde a 95,86% da pontuação máxima possível (232 pontos). Todos os indivíduos obtiveram escores totais superiores ao ponto de corte estabelecido pelo protocolo AMIOFE-E (152 pontos), com valores estimados variando entre 217 e 228 pontos. Em todos os domínios avaliados, 100% dos participantes apresentaram pontuações dentro da faixa de normalidade (≥ 50%), com destaque para os domínios de Mobilidade (98,86% da pontuação máxima) e Funções (96,3%). Observou-se maior variabilidade nas categorias de Mastigação (DP = 1,78) e Mandíbula –Aparência (DP = 1,83), refletindo diferenças individuais esperadas. Esses achados indicam preservação da funcionalidade orofacial na amostra estudada, conforme apresentado na Tabela 1.

**Tabela 1:** Avaliação Miofuncional Orofacial

**Item e Categoria**	**Pontuação máxima**	**Pontuações (N=10)** **Média ± DP**	**Mín – Máx**	**% da pontuação máxima**
Aparência/Postura	64	57,7 ± 3,95	53,75 – 61,65	90,16%
Face	12	9,8 ± 1,23	8,57 – 11,03	81,67%
Bochechas	8	7,6 ± 0,52	7,08 – 8,12	95,00%
Mandíbula	12	10±1,83	8,17 – 11,83	83,33%
Lábios	16	10,9 ±1,1	9,80 – 12,00	68,12%
Palato	8	7,8 ±0,42	7,38 – 8,22	97,50%
Língua	8	7,9 ±0,32	7,58 – 8,22	98,75%
Mobilidade	114	112,7 ±1,7	111,00-114,40	98,86%
Lábios	24	23,7 ±0,67	23,03 – 24,37	98,75%
Língua	36	36 ±0	36,00	100,00%
Mandíbula	30	29,4 ± 1,26	28,14 – 30,66	98,00%
Bochechas	24	23,6 ± 1,26	22,34 – 24,86	98,33%
Funções	54	52 ± 2, 26	49,74 – 54,26	96,30%
Respiração	4	3,9 ± 0,32	3,58 – 4,22	97,50%
Deglutição	28	27,6 ± 0,7	26,90 – 28,30	98,57%
Mastigação	22	20,5 ±1,78	18,72 – 22,28	93,18%
Total AMIOFE-E	232	222,4 ± 5,87	216,92 – 227,88	95,86%

**Legenda:** AMIOFE-E = Protocolo de Avaliação Miofuncional Orofacial com Escores Expandido; N = Número de participantes; DP = desvio padrão; Min = mínimo; Max = máximo; % = percentual calculado em relação à pontuação máxima possível por categoria/domínio

A Tabela 2 apresenta, simultaneamente, as medianas (IQR) das semanas 1 e 2 e os índices de confiabilidade intra-avaliador (ICC, EPM e DMD) para os quatro movimentos. Observou-se aumento significativo apenas para a lateralização direita (p = 0,047), enquanto elevação, lateralização esquerda e protrusão não diferiram entre as semanas (p ≥ 0,169). A confiabilidade variou de boa a excelente (ICC = 0,887–0,971), com menor EPM e DMD na lateralização direita (EPM = 0,86; DMD = 2,38) e maior na lateralização esquerda (EPM = 1,55; DMD = 4,30), indicando maior precisão para a lateralização direita.

**Tabela 2:** Medidas de pressão da língua (Semanas 1 e 2) e confiabilidade intra-avaliador (N = 10)

**Movimentos**	**Semana 1 Mediana (IQR) kPa**	**Semana 2 Mediana (IQR) kPa**	**Valor-p**	**ICC (IC95%)**	**EPM**	**DMD**	**Valor-p**
**Elevação**	21,76	23,21	0,169	0,9	1,45	4,05	< 0,001
	(0,01–26,19)	(21,99–25,39)		(0,623–0,975)			
**Lateralização D**	14,42	14,86	0,047*	0,96	0,86	2,38	< 0,001
	(12,06–19,43)	(13,26–20,58)		(0,803–0,990)			
**Lateralização E**	17,39	16,99	0,799	0,887	1,55	4,3	0,002
	(12,90–22,80)	(13,98–20,94)		(0,527–0,972)			
**Protrusão**	26,32	27,85	0,285	0,971	1,02	2,83	< 0,001
	(24,11–31,39)	(23,75–32,78)		(0,889–0,993)			

Teste Wilcoxon

* Valores significativos a P < 0,05

**Legenda:** IQR = intervalo interquartil; kPa = quilopascal; ICC = Coeficiente de Correlação Intraclasse; IC95% = Intervalo de Confiança de 95%; EPM = Erro Padrão da Medida; DMD = Diferença Mínima Detectável

A Figura 3 mostra através da análise de *Bland-Altman* que houve boa concordância entre as semanas 1 e 2 para todos os movimentos, com diferenças médias próximas de zero. Observou-se maior variabilidade na elevação (Média da diferença: 1,18 kPa, DP: 2,68) e na protrusão (Média da diferença: 0,84 kPa, DP: 1,91), enquanto a lateralização direita apresentou menor variabilidade (Média da diferença: 0,98 kPa, DP: 1,48) e a lateralização esquerda mostrou maior dispersão (Média da diferença: 0,15 kPa, DP: 2,93).

**Figura 3:** Representações gráficas do diagrama de Bland–Altman para as medições de pressão da língua nos movimentos de elevação, lateralização direita, lateralização esquerda e protrusão

## DISCUSSÃO

Este estudo avaliou a reprodutibilidade das medidas de pressão da língua em indivíduos adultos utilizando um dispositivo experimental e de baixo custo. A confiabilidade intra-avaliador mostrou-se boa a excelente, sugerindo que as medições podem ser utilizadas na prática clínica.

O dispositivo utilizado neste estudo, além de apresentar boa estabilidade e permitir registro em tempo real, facilitando a avaliação e *feedback* para o participante, foi desenvolvido com componentes de baixo custo e acessíveis no mercado nacional. Detalhes do desenvolvimento e do custo estimado (aproximadamente US$ 49) estão descritos em Almeida et al.^(14)^, o que sugere sua viabilidade para uso clínico e domiciliar, inclusive em contextos com recursos limitados.

Em comparação com dispositivos tradicionais, como o IOPI, amplamente utilizado para a mensuração da pressão lingual, este dispositivo apresenta maior versatilidade. O IOPI é tradicionalmente empregado na avaliação da pressão de elevação^(20)^, mas estudos indicam que pode ser adaptado para medições de lateralização, ampliando sua aplicabilidade^(16,21)^. Neste estudo, seguimos os procedimentos com o IOPI descritos por Clark *et al*.^(8)^, realizando medições de elevação, lateralização e protrusão (com o bulbo acoplado à espátula para as medidas direcionais), o que permite uma análise mais abrangente da funcionalidade lingual^(8)^. No presente estudo, os coeficientes de Correlação Intraclasse para a pressão da língua foram semelhantes aos relatados previamente^(13,22)^, variando de 0,72 a 0,97. Esses achados reforçam a confiabilidade e reprodutibilidade de dispositivos objetivos na avaliação da pressão lingual. O estudo de Diaz-Saez et al.^(22)^ realizado com a população da Espanha, avaliou um dispositivo para mensuração da pressão da língua e relatou boa a excelente confiabilidade intra e inter-avaliador, com valores de ICC superiores a 0,80, reforçando a viabilidade do uso desses instrumentos na prática clínica. Araújo et al.^(13)^ analisaram a reprodutibilidade do Forling, um instrumento portátil desenvolvido por pesquisadores brasileiros para medir a força axial da língua, e observaram coeficientes de correlação intraclasse aceitáveis, além de variação dentro da margem desejável, indicando sua confiabilidade para uso clínico e em pesquisas. Embora a maioria das medições deste estudo tenha permanecido inalterada entre os testes, observou-se um aumento significativo na lateralização direita na segunda semana. Como os participantes passaram por um processo prévio de familiarização, essa diferença pode estar associada aos ajustes motores e refinamento neuromuscular ao longo das sessões experimentais^(23)^. A neuroplasticidade permite ajustes no controle motor, favorecendo a aprendizagem motora^(24)^. Estudos indicam que movimentos repetitivos da língua podem induzir neuroplasticidade e aprimorar a coordenação intermuscular^(25)^. O estudo de Kamarunas et al.^(23)^ destaca que a otimização do recrutamento motor e da coordenação pode aumentar a força muscular. Esse efeito poderia explicar a melhora na lateralização direita, alinhando-se aos achados de Araújo et al.^(13)^ que observaram aumento progressivo da força ao longo das medições, atribuindo também à adaptação ao procedimento de avaliação, especialmente entre a primeira e segunda sessão experimental.

A confiabilidade intra-avaliador foi considerada de boa a excelente, demonstrando consistência entre as semanas 1 e 2. No entanto, a lateralização esquerda apresentou maior variabilidade, possivelmente devido a um indivíduo com valores discrepantes, conforme evidenciado pela análise de *Bland-Altman*. Esse indivíduo foi identificado no AMIOFE-E, e algumas alterações em itens como mastigação e relação mandibular podem ter contribuído para a variação da força da língua entre a primeira e a segunda sessão experimental (Figura 3). Isso sugere que fatores estruturais ou funcionais, como a oclusão dentária e a articulação temporomandibular (ATM), podem influenciar os movimentos da língua. O estudo de Yu e Gao^(26)^ demonstrou que a distribuição da pressão da língua está relacionada à forma do arco dentário e à oclusão. Os autores observaram que a pressão da língua contra o palato é influenciada pela largura e pelo comprimento do arco dentário, impactando a estabilidade da mordida e a funcionalidade orofacial. Além disso, Diaz-Saez et al.^(27)^ identificaram diferenças significativas na pressão da língua entre mulheres assintomáticas e aquelas com disfunção temporomandibular (DTM), evidenciando a relevância dessa avaliação clínica. Nesse estudo, foi observada uma redução média de aproximadamente 30% na pressão da língua em todas as direções no grupo com DTM, reforçando a necessidade de considerar a pressão da língua na prática clínica. Os resultados deste estudo mostraram que as medidas de pressão da língua foram inferiores às relatadas em pesquisas que utilizaram dispositivos comerciais^(9,10)^. Essa diferença pode estar associada às particularidades dos dispositivos, que variam em sensibilidade, calibração e área de contato, bem como às características da população avaliada. Estudos indicam que os valores reportados podem ser influenciados pelo método de aferição, pelo posicionamento do sensor e pelo protocolo de medição^(28)^. Além disso, fatores como sexo, idade e familiarização com o teste podem impactar os resultados, sendo observado que homens tendem a apresentar maior pressão lingual, enquanto os movimentos de lateralização e protrusão são menos explorados na literatura^(29)^.

A estabilidade do sensor também pode afetar a medição da pressão da língua, especialmente em movimentos de lateralização e protrusão. Solomon e Clark^(30)^ demonstraram que a adição de uma superfície antiderrapante ao sensor resultou em valores significativamente maiores de pressão máxima nessas direções, sugerindo que o deslizamento da língua pode comprometer a precisão dos resultados. Isso destaca a importância de considerar o tipo de suporte utilizado na mensuração da pressão lingual, especialmente em estudos comparativos entre diferentes dispositivos e metodologias.

É importante destacar que este estudo apresenta algumas limitações. Foram avaliadas apenas as pressões máximas de elevação, lateral e protrusão da língua, sem incluir a pressão máxima posterior, o que restringe uma visão completa da funcionalidade lingual em diferentes direções de movimento. Além disso, a amostra foi composta exclusivamente por adultos saudáveis, o que não permite extrapolar diretamente os resultados para populações com alterações orofaciais ou condições clínicas específicas. Futuros estudos devem contemplar diferentes direções de movimento e incluir grupos clínicos para ampliar a generalização dos achados.

Os achados deste estudo podem impactar a prática clínica da motricidade orofacial, ao demonstrar que o dispositivo experimental é uma ferramenta acessível e de baixo custo para a avaliação da pressão lingual. Futuros estudos devem investigar sua aplicabilidade em diferentes contextos clínicos, ampliando as possibilidades de uso.

## CONCLUSÃO

Este estudo demonstrou que a mensuração da pressão da língua, com o dispositivo proposto, em indivíduos adultos apresenta boa a excelente reprodutibilidade. A protrusão foi o movimento de maior precisão, enquanto a lateralização esquerda mostrou maior variabilidade, possivelmente devido a fatores estruturais e funcionais. A alta confiabilidade intra-avaliador indica que o dispositivo pode ser uma ferramenta útil para avaliações objetivas da pressão da língua. Além disso, seu baixo custo favorece a incorporação na prática fonoaudiológica, ampliando as possibilidades de avaliação e acompanhamento clínico.
